# Understanding determinants of parental HPV vaccine hesitancy under a municipal free vaccination program in Guangzhou, China

**DOI:** 10.1093/heapol/czaf087

**Published:** 2025-11-10

**Authors:** Anqi Li, Peiqi Wang, Jiayue Li, Weilin Chen, Jinghui Chang

**Affiliations:** School of Health Management, Southern Medical University, Guangzhou 510515, China; School of Health Management, Southern Medical University, Guangzhou 510515, China; School of Health Management, Southern Medical University, Guangzhou 510515, China; School of Health Management, Southern Medical University, Guangzhou 510515, China; School of Health Management, Southern Medical University, Guangzhou 510515, China

**Keywords:** HPV vaccination, vaccine hesitancy, free immunization policy, health equity, China

## Abstract

Despite efforts to promote HPV vaccination, coverage remains suboptimal in China. Following Guangzhou's 2022 free HPV vaccination program for girls aged 9–15, a cross-sectional survey was conducted from May to August 2024 among 411 parents of eligible girls in Guangzhou. The questionnaire was developed based on the supply–demand alignment theory. Vaccine Hesitancy Scale and Family Health Scale-Short Form were administered. Generalized linear regression identified factors associated with hesitancy. Overall, 10.7% of parents exhibited high hesitancy. Key determinants included occupation [farmers: β = −3.61, 95% CI = (−6.88, −0.34)], preference for imported over domestic vaccines [β = −1.65, 95% CI = (−3.10, −0.12)]. Higher family health scores [β = 0.25, 95% CI = (0.16, 0.33)], moderate child health status [β = 1.24, 95% CI = (0.10, 2.38)], and satisfaction with community healthcare centers (CHCs) [β = 0.05, 95% CI = (0.02, 0.07)] were less hesitant. Paradoxically, longer CHC wait times (>1 hour) [β = 2.29, 95% CI = (0.27, 4.31)] and difficulty accessing information [β = 2.80, 95% CI = (0.33, 5.27)] correlated with lower hesitancy. The results suggest potential policy-driven tolerance. Besides, this emphasizes the critical need for enhanced service quality in CHCs, targeted health education, and confidence building in national vaccines. These insights offer potential guidance for implementing complementary strategies to achieve equitable HPV vaccine coverage.

Key Messages

- Persistent disparities in HPV vaccine hesitancy exist among specific groups. Parental occupation (e.g. farmers) and distrust in domestically produced vaccines were key determinants of hesitancy, indicating the need for further context-specific research and potential targeted interventions in rural and low-trust communities.
- Service quality and accessibility of vaccination spots significantly influence vaccine acceptance. Higher satisfaction with community healthcare centers and accessible information reduced hesitancy, emphasizing the importance of improving healthcare infrastructure and transparent communication strategies.
- Free vaccination policies show promise but require complementary strategies. While Guangzhou's free HPV vaccination policy achieved relatively low hesitancy, equitable coverage demands tailored approaches, such as localized health education, subsidies for vulnerable groups, and partnerships with schools and communities.

## Introduction

Cervical cancer is the fourth most common cancer worldwide and one of the leading causes of female mortality (WHO 2023). In 2020, China reported ∼110 000 new cases of cervical cancer and 59 000 deaths, accounting for 18% of global cervical cancer deaths (Cao et al. 2021, Sung et al. 2021). Human papillomavirus (HPV) infection is the primary etiological factor for cervical cancer, with high-risk HPV types 16 and 18 responsible for over 70% of cervical cancer and precancerous lesions, and 90.8% of cervical cancer patients being infected with high-risk HPV (Li et al. 2019). Vaccination against HPV is an effective measure for preventing cervical cancer and precancerous lesions (WHO 2023). The World Health Organization (WHO) recommends that girls aged 9 to 14, who have not yet initiated sexual activity, be prioritized for HPV vaccination. The “Global Strategy to Accelerate the Elimination of Cervical Cancer” sets the goal of achieving 90% coverage with HPV preventive vaccination among girls under 15 years of age through a comprehensive immunization program (WHO 2023, 2009).

Guangdong Province in China has been actively working to align with this global strategy, following the “Promoting Healthy China Action” and “Healthy Guangdong” policies (Province 2020), implementing cervical cancer prevention and control initiatives. However, the current HPV vaccination coverage in China is not optimistic. The HPV vaccination coverage in China remains low. Between 2018 and 2020, only 2.24% of eligible women aged 9–45 years were vaccinated (Song et al. 2021). In contrast, countries such as the United Kingdom, the United States, and Australia have implemented comprehensive free HPV vaccination programs for girls as early as 2008 (Chow et al. 2019, NHS 2023, White et al. 2024) and have gradually expanded the target population to include eligible boys (Brown et al. 2010, Chow et al. 2019), thereby further increasing vaccine coverage. Some provinces and cities in China have gradually implemented free HPV vaccination programs for eligible girls after 2022 (Province 2022, 2023, Xizang 2023). In Guangzhou city, a free HPV vaccination program for eligible girls was launched in January 2022 (Municipality 2022a), with districts officially initiating related efforts by September of the same year. Health and education departments have worked together to implement vaccinations from September to November annually, with schools playing a key role in promoting and facilitating vaccinations (Municipality 2022b). The goal is to reach 90% HPV vaccination coverage among girls under 15 by 2030. However, in certain regions of China, such as Anhui Province, the HPV vaccine has yet to be incorporated into national or provincial immunization programs.

Although prior research anticipated that reducing HPV vaccine costs would increase parental willingness in China (Tan et al. 2024). Studies reveal persistent non-financial barriers even under free/subsidized policies. For instance, most female healthcare workers maintained their vaccination attitudes despite subsidy scenarios (Lu et al. 2022), while parents consistently prioritized 9-valent vaccines (self-paid) for their daughters over lower-cost options for themselves (Zhou et al. 2024).

In 2015, the Strategic Advisory Group of Experts on Immunization (SAGE) of the WHO defined vaccine hesitancy as a continuum of attitudes that ranges between absolute acceptance of vaccines and absolute refusal or delay in receiving vaccines, despite the availability of vaccination services (MacDonald 2015). It represents a dynamic and evolving stance within the intermediate range between complete acceptance and complete refusal of vaccines (Shen et al. 2019). In 2019, the WHO identified vaccine hesitancy as one of the top 10 global health threats (WHO 2019). Research on vaccine hesitancy is more extensive in foreign countries, and in 2015, SAGE developed a matrix categorizing determinants of vaccine hesitancy into three main groups: contextual influences, individual/group influences, and vaccine-specific factors (MacDonald 2015).

Research on vaccine hesitancy in China has emerged more recently, often relying on international theories and measurement tools. In the early stages, Shi et al. (2019) and Xiong et al. (2019) explored the global anti-vaccine movement and factors influencing vaccine hesitancy. Currently, vaccine hesitancy research in China primarily has focused on vaccines for coronavirus (Liu et al. 2021b), influenza (Zhao et al. 2021), and human papillomavirus (Song et al. 2023), with populations including caregivers, university students, healthcare workers, and the elderly (Wei and Fu 2019, Liu et al. 2021c, Zhao et al. 2021, Song et al. 2023). Theories like the Knowledge Attitudes and Practices (KAP) model (Ma et al. 2018), Health Belief Model (Mercadante and Law 2021), and Theory of Planned Behavior (Dubé et al. 2018) have been applied. Existing research indicates that vaccine hesitancy is influenced by factors like media environment, historical context, religion, culture, gender, socioeconomic status, and geographical barriers (Liu et al. 2021a).

Concerning the HPV vaccines, Wu et al. (2023), based on the 3C model framework, found that vaccine hesitancy can be influenced by factors such as perceived vaccine effectiveness, inadequate self-awareness, insufficient or incorrect knowledge about the vaccine, and the complexity of the vaccination process. Both domestic and international research have increasingly focused on the role of the family in vaccine uptake. Studies have shown that family health functioning is related to children's utilization of health services (Van Fossen et al. 2022). Family support is associated with lower levels of vaccine hesitancy (Rustagi et al. 2022). Xu et al. (2020) found that maternal vaccine hesitancy is positively influenced by family health education. However, research in China on this subject is still relatively limited. Meanwhile, globally, most studies and strategies addressing vaccine hesitancy have primarily focused on the vaccine recipients. In the social context of China, “vaccine immunization programs” have emerged as a more significant strategy to address hesitancy (Yang et al. 2019).

Against this background, it is hypothesized that even under the municipal free vaccination program, factors concerning vaccination services and family, like service quality factors and family health functioning, would influence parental HPV vaccine hesitancy. This study investigates the willingness of parents in Guangzhou to vaccinate their 9- to 15-year-old daughters under the free HPV vaccine policy, ultimately providing insights to improve HPV vaccine uptake among eligible populations.

## Methods and materials

### Participants

The cross-sectional survey targeted parents or legal guardians of female children aged 9–15 years residing in Guangzhou, Guangdong Province, China. The surveys were mainly carried out in schools and community healthcare centers (CHCs). Participation was voluntary and based on informed consent. Eligibility screening was implemented through an initial question: “Do you have a daughter aged 9–15 years?” Respondents answering “no” were automatically excluded from the survey. The inclusion criteria of parents were as follows: (1) Aged 18 and above; (2) Biological or legal guardian of at least one girl aged 9–15 years; (3) Current residency in Guangzhou. Exclusion criteria were the following: (1) Individuals with diagnosed mental health disorders; (2) Persons with cognitive impairments affecting decision-making capacity.

Key parameters of sample size as follows. Confidence level (1−α) was set as 95%. Margin of error (e) was 0.05, balancing precision and feasibility. Expected proportion (*P*) was 67.6% representing a conservative assumption for a non-expanded program on immunization (non-EPI) vaccine hesitancy reported according to previous research in the Chinese adults (Wang et al. 2021a). Target population (N): Estimated from 2023 census data as 18 827 000 people in Guangzhou, representing a conservative assumption for parents of girls. Response rate adjustment was 80%, accounting for potential non-participation. The minimum sample size was calculated using the standard formula for proportions:


$$n=\frac{{Z_{\left(1-\alpha \right)}}^{2}\times p\times (1-p)}{e^{2}}$$

A finite population correction was applied given the known target population size:


$$n_{adjusted}=\frac{n}{1+\frac{\left(n-1\right)}{N}}$$

For *N* = 18 827 000: *n*(adjusted) = 336/(1+ 383/18 827 000) ≈ 336 (negligible adjustment). To compensate for an anticipated 80% response rate, the final sample size was inflated to 420.

Finally, 450 samples were collected and 411 samples were valid, representing a valid response rate of 91.33%.

### Procedure

**Figure 1.**
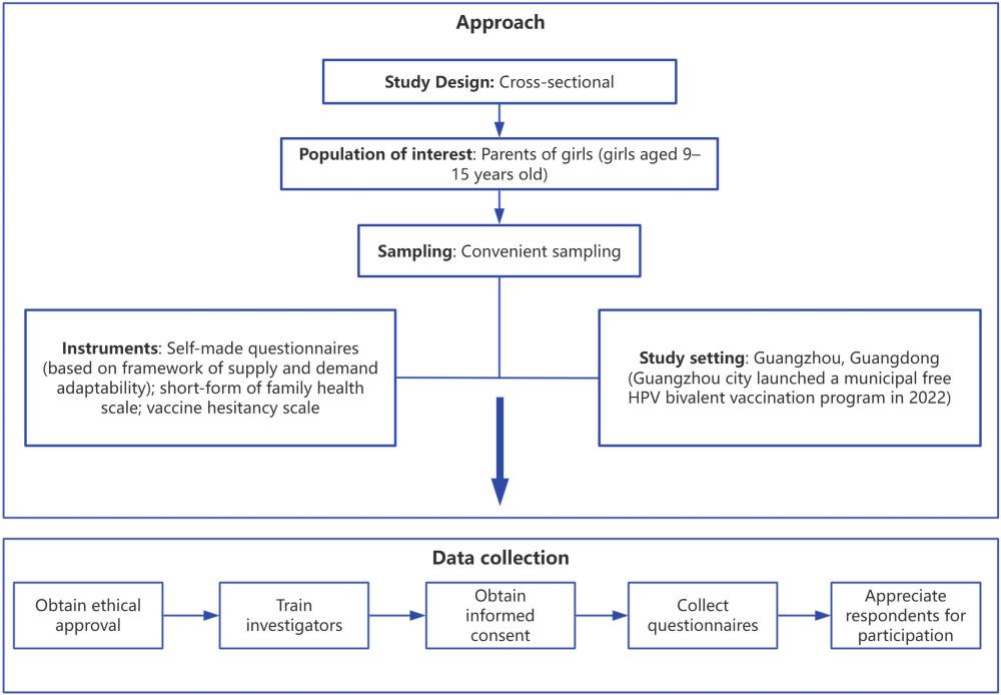
Schematic representation of the research approach.

From May to August 2024, a convenient sampling method was employed to select eligible parents of girls in Guangzhou. The survey was conducted through an online platform (QuestionStar platform) or via printed questionnaires. Questionnaires were distributed through online methods, including group messaging and social media posts, as well as face-to-face interviews (Fig. 1).

### Tool

#### Self-made Questionnaires

The study used a self-designed questionnaire to collect data on participants' demographic characteristics, vaccine knowledge, and the alignment between the supply and demand of HPV vaccination services from both the provider and recipient perspectives. The questionnaire was grounded in the theoretical framework of supply and demand adaptability to examine the alignment of HPV vaccination services among parents of 9- to 15-year-old girls in Guangzhou. The supply–demand alignment theory focuses on the “mismatch” between the actual needs of the target population or service recipients and the services provided. It analyses various forms of this “mismatch” to evaluate whether the services offered by social policies adequately meet social needs and assess the allocation of service resources. The theory consists of four dimensions: relevance, appropriateness, accessibility, and quality (Cook 2007). This theory has been applied in health research in China, such as evaluating health resource allocation for children (Liu et al. 2022).

The study assessed the following dimensions: relevance, which refers to whether the government-provided free vaccination service for domestically produced bivalent HPV vaccine meets the actual needs of families; appropriateness, which refers to whether the free HPV vaccination service considers the diversity and complexity of children's vaccination needs; accessibility, which measures the acceptability of the distance to the HPV vaccination service provided by parents of children, which indicated CHCs in the study (Supplementary Appendix Table S1).

#### Short-form of Family Health Scale

Short-form of the Family Health Scale (FHS-SF) is used to measure the functional health of families. It consists of 10 items, with items 6, 9, and 10 reverse-scored. The scale items are scored using a 5-point Likert scale (completely disagree, disagree, uncertain, agree, completely agree). The Cronbach's α coefficient for the scale is 0.83, indicating good internal consistency. The model fit indices show a good fit: *x*^2^/degrees of freedom (df) = 4.28 < 5; goodness-of-fit index (GFI) = 0.98 > 0.85; normed fit index (NFI) = 0.97 > 0.90; relative fit index (RFI) = 0.95 > 0.90; root mean square error of approximation (RMSEA) = 0.07 < 0.08. Therefore, the scale demonstrates good reliability and validity.

In this study, the Cronbach's α coefficient for the Family Health Scale was 0.793.

#### Vaccine Hesitancy Scale

The Vaccine Hesitancy Scale (VHS), a 10-item scale, was developed by the WHO's SAGE Working Group (Larson et al. 2015) was used to measure vaccine hesitancy as an attitude. Each item of the VHS is rated on a 5-point Likert scale (completely disagree, disagree, uncertain, agree, completely agree), with scores ranging from 1 to 5. Reverse scoring is applied to items 5, 9, and 10. A lower total score on the scale indicates a higher level of vaccine hesitancy, with a cutoff of ≤30 defining “high hesitancy” (Kempe et al. 2020, Wang et al. 2021b).

In this study, the VHS showed good internal consistency with a Cronbach's α coefficient of 0.733.

### Quality control

Both the online and offline versions of the questionnaire clearly outlined the purpose, methods, and data usage of the survey. To ensure research quality, all participants were required to review reading materials on the HPV vaccine policy in Guangzhou before completing the questionnaire. Data entry was performed using Excel software, and a dedicated team member verified the data to ensure its authenticity and reliability. Upon completion of the survey, data screening was conducted. Disqualified questionnaires were excluded based on the following criteria: (i) incomplete responses, (ii) clear logical inconsistencies, and (iii) response times of less than 60 seconds.

For the field survey and online survey, staff were assigned to monitor the progress of the online survey daily. After each day’s data collection, a dedicated staff member reviewed the surveys for completeness, accuracy, and authenticity. The platform's user identification system was used to confirm the authenticity, uniqueness, and representativeness of participants. Offline survey staff underwent training and assessments to ensure consistency and accuracy.

### Statistical analysis

Data analysis was conducted using SPSS 27.0. Cronbach's α was calculated to assess the internal consistency of the scales. The Kolmogorov–Smirnov test was used to determine the normality of continuous variables. Continuous variables were found to have a non-normal distribution and are presented as medians and interquartile ranges (IQR). Categorical variables are reported as numbers and percentages. The relationship between variables and the VHS score was analyzed using a univariate generalized linear model. Subsequently, linear regression models with generalized estimating equations were used to identify factors influencing HPV vaccine hesitancy. Regression coefficients (β) and 95% confidence intervals (CIs) were calculated. Statistical significance was set at α = 0.05.

## Results

### Basic characteristics of parents and comparison

A total of 450 questionnaires were collected, of which 411 were valid (91.33% valid response rate). The majority of parents were aged 36–45 years. Of the respondents, most participants were female (69.82%) and married (92.21%).

The study examined vaccine hesitancy among parents of girls, considering various factors such as gender, education, occupation (Table 1), previous unsatisfactory vaccination experiences, trust in domestic versus imported vaccines, the health status of the girls, attention to HPV vaccine-related information, prior vaccine refusal (*P* < 0.05). Additionally, the study explored the impact of factors like waiting time at CHCs, and difficulty in accessing information from schools or CHCs, the adequacy of HPV vaccine publicity in meeting information needs, proximity to vaccination locations, understanding of the vaccination process, the provision of platforms for reservations and inquiries, and soundness of the CHC system (Supplementary Appendix Table S2).

**Table 1.** Comparison of parents’ VHS scores with different characteristics (*n* = 411).

Variables	Groups	*N* (%)	M (P25, P75)	β (95% CI)	*P* value
**Gender**	Male	127 (30.17)	36.00 (32.00, 38.75)	Reference	N/A
	Female	287 (69.82)	36.00 (36.00, 40.00)	1.248 (0.141, 2.354)	0.027*
**Age group**					
	≤35	61 (14.84)	36.00 (34.00, 40.00)	Reference	N/A
	36–45	272 (66.18)	36.00 (34.00, 39.00)	−0.618 (−2.083, 0.848)	0.409
	>46	78 (18.97)	36.50 (33.00, 39.25)	−0.658 (−2.426, 1.111)	0.466
**Marriage states**					
	Married	379 (92.21)	36.00 (33.00, 39.00)	Reference	N/A
	Others	32 (7.78)	36.00 (29.00, 41.00)	0.429 (−1.477, 2.335)	0.659
**Children numbers**					
	1	175 (42.57)	36.00 (34.00, 40.00)	Reference	N/A
	2	189 (45.98)	36.00 (33.00, 39.00)	−1.112 (−1.19, 0.972)	0.839
	≥3	47 (11.43)	36.00 (35.00, 39.00)	0.940 (−0.758, 2.639)	0.278
**Education**					
	Primary school and below	20 (4.86)	33.50 (30.00, 37.00)	Reference	N/A
	Middle school/high school/vocational school	142 (34.55)	36.00 (33.25, 38.75)	3.085 (0.635, 5.534)	0.014*
	Undergraduate or college	218 (53.04)	36.00 (33.00, 41.00)	3.220 (0.824, 5.617)	0.008*
	Postgraduate or above	31 (7.54)	37.00 (34.00, 39.00)	3.968 (1.026, 6.910)	0.008*
**Occupation**					
	Enterprise/business/service industry	174 (42.33)	36.00 (34.00, 38.00)	Reference	N/A
	Government civil servants	35 (8.51)	37.00 (35.00, 40.00)	1.119 (−0.771, 3.009)	0.246
	Worker	36 (8.75)	36.00 (34.00, 40.75)	0.695 (−1.173, 2.563)	0.466
	Farmer	9 (2.18)	31.00 (29.00, 32.50)	−5.471 (−8.959, −1.984)	0.002*
	Public institution employee	81 (19.70)	36.00 (34.00, 40.00)	−0.237 (−1.609, 1.136)	0.735
	Others	76 (18.49)	36.00 (32.25, 40.00)	−0.296 (−1.699, 1.107)	0.679
**Family type**					
	Core family	252 (61.31)	36.00 (34.00, 39.75)	Reference	N/A
	Nuclear family	101 (24.57)	36.00 (33.00, 39.00)	−0.878 (−2.093, 0.337)	0.157
	Other family	58 (14.11)	37.00 (32.00, 40.00)	−1.019 (−2.521, 0.484)	0.184
**Household registration**					
	Rural household registration	134 (32.60)	36.00 (33.00, 39.00)	Reference	N/A
	Urban household registration	277 (67.39)	36.00 (33.00, 39.00)	−0.733 (−1.821, 0.354)	0.186
**Monthly income**					
	<5000 CNY	88 (21.41)	36.50 (32.00, 40.00)	Reference	N/A
	5000–9999 CNY	179 (43.55)	36.00 (34.00, 39.00)	0.432 (−0.915, 1.779)	0.530
	10 000–14 999 CNY	80 (19.46)	36.00 (33.00, 39.00)	0.139 (−1.460, 1.737)	0.865
	>15 000 CNY	64 (15.57)	36.00 (34.00, 39.00)	−0.183 (−1.883, 1.516)	0.883

*P < 0.05.

### Vaccine hesitancy scores among parents of girls

The median VHS score of parents was 36, and 44 parents (10.7% of the surveyed population) were highly hesitant (VHS score ≤30) (Table 2).

**Table 2.** Vaccine Hesitancy Scale and its subscale total scores among parents of girls (*n* = 411).

Vaccine hesitancy scale	M (P25, P75)
**Total scores**	**36.07** (**33, 39)**
Q1. HPV vaccines are important for my child's health.	4.18 (4.00, 5.00)
Q2. HPV vaccines are effective.	4.11 (4.00, 5.00)
Q3. Having my child vaccinated is important for the health of others in my community	3.87 (3.00, 5.00)
Q4. All HPV vaccines offered by the government programme in my community are beneficial.	3.97 (4.00, 5.00)
Q5. HPV vaccines carry more risks than older vaccines.	3.14 (3.00, 4.00)
Q6. The information received about HPV vaccines from the vaccine programme is reliable and trustworthy.	3.92 (3.00, 4.00)
Q7. Getting HPV vaccines is a good way to protect my child/children from disease.	3.93 (3.00, 5.00)
Q8. Generally I do what my doctor or health care provider recommends about HPV vaccines for my child/children.	4.06 (4.00, 5.00)
Q9. I am concerned about serious adverse effects of HPV vaccines.	2.33 (2.00, 3.00)
Q10. My child/children does or do not need HPV vaccines for diseases that are not common anymore.	2.57 (2.00, 3.00)

### Factors influencing HPV vaccine hesitancy among parents of girls

Multiple generalized linear regression analysis with generalized estimating equations was conducted with the total score of the VHS score as the dependent variable. The significant factors identified in the univariate analysis were included in the regression model.

To better address the relationships among variables, we have performed several analyses by grouping variables into three models, demographic and social economic factors (Supplementary Appendix Table S3), demographic and social economic factors plus health state indicators (Supplementary Appendix Table S4), demographic and social economic factors plus experiences and preferences (Supplementary Appendix Table S5). The key results revealed that (included all significant variables from demographic and social economic factors, health state indicators, vaccine attitude and supply and demand adaptability of HPV vaccine service) parents who worked as farmers [β = −3.61, 95% CI = (−6.88, −0.34)] and those who with low confidence in the Chinese-made vaccine, preferring the imported vaccine [β = −1.65, 95% CI = (−3.10, −0.12)], exhibited higher vaccine hesitancy. Conversely, parents of girls in moderate health status [β = 1.24, 95% CI = (0.10, 2.38)] show lower vaccine hesitancy. Additionally, parents who had experienced waiting times of more than one hour at CHCs [β = 2.29, 95% CI = (0.27, 4.31)], parents who reported “very difficult” in accessing relevant information at CHCs [β = 2.80, 95% CI = (0.33, 5.27)], parents who had high levels of satisfaction with the CHCs [β = 0.05, 95% CI = (0.02, 0.07)], and those reported high family health scores [β = 0.25, 95% CI = (0.16, 0.33)] were less hesitant to vaccinate their daughters (Table 3).

**Table 3.** Generalized linear regression of factors associated with the HPV vaccine hesitancy among parents of girls (*n* = 411).

Variables		β (SE)	95% CI	X^2^ (df)	*P* value
**Gender** (reference: male)					
	Female	0.24 (0.52)	(−0.78, 1.26)	0.22 (1)	0.64
**Education** (reference: primary school and below)					
	Middle school/high school/vocational school	2.04 (1.17)	(−0.25, 4.33)	3.05 (1)	0.08
	Undergraduate or college	1.7 (1.2)	(−0.65, 4.04)	2 (1)	0.16
	Postgraduate or above	2.01 (1.45)	(−0.83, 4.86)	1.92 (1)	0.17
**Occupation category** (reference: enterprise/business/service industry)					
	Government civil servants	1.23 (0.91)	(−0.54, 3.01)	1.85 (1)	0.17
	Worker	0.74 (0.89)	(−1.01, 2.48)	0.68 (1)	0.41
	Farmer	−3.61 (1.67)	(−6.88, −0.34)	4.68 (1)	0.03*
	Public institution employee	0.78 (0.67)	(−0.54, 2.09)	1.34 (1)	0.25
	Others	−0.14 (0.64)	(−1.39, 1.12)	0.05 (1)	0.83
**Prior vaccine refusal** (reference: No)					
	Yes	1.16 (0.65)	(−0.12, 2.43)	3.17 (1)	0.08
**Previous unsatisfactory vaccine experience** (reference: No)					
	Yes	−0.11 (0.59)	(−1.26, 1.04)	0.04 (1)	0.85
**Health state of girls** (reference: Good)					
	Moderate	1.24 (0.58)	(0,10, 2.38)	4.56 (1)	0.03*
	Poor	−0.96 (1.48)	(−3.87, 1.95)	0.42 (1)	0.52
**Trust in domestic versus imported vaccines** [reference: High level of trust (no preference for imported vaccines)]					
	Mostly trust (some concerns about certain vaccines)	−0.58 (0.58)	(−1.72, 0.57)	0.98 (1)	0.32
	Not very trusting (preference for imported vaccines)	−1.65 (0.76)	(−3.10, −0.12)	4.46 (1)	0.04*
	Very distrustful (no preference for domestically produced vaccines)	0.31 (1.72)	(−3.06, 3.68)	0.03 (1)	0.86
**Attention to HPV vaccine-related information** (reference: lack of attention)					
	Limited attention	−1.14 (0.62)	(−2.35, 0.07)	3.39 (1)	0.07
	Moderate attention	−0.6 (0.74)	(−2.05, 0.84)	0.67 (1)	0.41
	Significant attention	−0.5 (0.97)	(−2.4, 1.4)	0.27 (1)	0.61
					
**Adequacy of HPV vaccine publicity in meeting information needs** (reference: no)					
	Yes	−0.34 (0.55)	(−1.41, 0.73)	0.40 (1)	0.53
**Proximity to vaccination locations** (reference: no)					
	Yes	−0.99 (0.57)	(−2.11, 0.13)	2.99 (1)	0.08
**Waiting time in CHC ^a^** (reference: <10 minutes)					
	10–29 minutes	1.69 (0.9)	(−0.07, 3.44)	3.54 (1)	0.06
	30–59 minutes	0.98 (0.89)	(−0.77, 2.72)	1.2 (1)	0.27
	>1 hour	2.29 (1.03)	(0.27, 4.31)	4.94 (1)	0.03*
**Difficulty in accessing information from the school** (reference: very easy)					
	Relatively easy	−1.13 (0.71)	(−2.52, 0.26)	2.53 (1)	0.11
	Relatively difficult	−0.65 (0.84)	(−2.29, 0.99)	0.61 (1)	0.44
	Very difficult	−0.48 (1.25)	(−2.93, 1.97)	0.15 (1)	0.70
**Difficulty in accessing information from CHC ^a^** (reference: very easy)					
	Relatively easy	−1.36 (0.67)	(−2.68, −0.04)	4.08 (1)	0.04*
	Relatively difficult	−0.03 (0.85)	(−1.69, 1.63)	0 (1)	0.97
	Very difficult	2.80 (1.26)	(0.33, 5.27)	4.94 (1)	0.03*
**Understanding of the vaccination process** (reference: no)					
	Yes	0.19 (0.58)	(−0.94, 1.32)	0.11 (1)	0.74
**The soundness of the management system in CHC^a^** (reference: No)					
	Yes	−0.66 (0.52)	(−1.68, 0.37)	1.58 (1)	0.21
**CHC^a^ satisfaction score**		0.05 (0.01)	(0.02, 0.07)	11.25 (1)	<0.01*
**Family health score**		0.25 (0.04)	(0.16, 0.33)	31.13 (1)	<0.01*

^a^Community healthcare center (CHC).

^*^ *P* < 0.05.

## Discussion

### General findings on parents with high vaccine hesitancy

The results of this study revealed that 10.7% parents of girls classified as having a high level of vaccine hesitancy (VHS score ≤ 30). This rate is lower than the reported vaccine hesitancy of 19.3% among parents in Los Angeles (Tsui et al. 2023), and 23% among national parents in the United States (Szilagyi et al. 2020). However, it is still higher than the high hesitancy rate for non-EPI vaccines in coastal and more developed area of China, such as Jiangsu Province, where 7.7% of parents were highly hesitant about non-EPI vaccines (Yang et al. 2022). In comparison to a nationwide vaccine hesitancy survey conducted in China from February to April 2021, which found a rate of 27.4% girls were willing to vaccinate themselves (Zhang et al. 2021a), the hesitancy rate for the HPV vaccine in this study was also higher. These findings suggest that, while the HPV vaccine has a relatively high hesitancy rate among non-EPI vaccines, there is still room for improvement in reducing vaccine hesitancy among parents of girls in Guangzhou.

### Targeted HPV vaccine education and policy interventions for farmers

Despite the implementation of free HPV vaccination policies, parents in farming occupations continue to exhibit higher levels of hesitancy compared to parents in other professions. This is consistent with findings from a vaccine hesitancy survey in Jiangsu, China, where agricultural workers, including those in forestry, fisheries, water conservancy, and animal husbandry, were more hesitant about non-EPI vaccines, including the HPV vaccine (Yang et al. 2022). In China, the majority of farmers are characterized by low educational attainment and income levels (2023). However, studies have presented varying perspectives on the association between socioeconomic status and vaccine hesitancy. Unlike in high-income countries, there was no consistent association between participants’ education level and vaccine hesitancy in middle- and low-income countries (Wagner et al. 2019). Furthermore, research on HPV vaccine awareness in China have shown that rural areas have lower levels of HPV vaccine knowledge compared to urban regions (Zhao et al. 2022) and significantly lower than those in the United States, the United Kingdom, and Australia (Marlow et al. 2013, Zhao et al. 2022). Farmers represent a critical group during the policy implementation process in China, and targeted HPV vaccine promotion and education efforts are essential to address vaccine hesitancy in rural and farming communities.

### Building trust in domestically produced HPV vaccines

This study found that parents of girls with moderate health status exhibited lower levels of vaccine hesitancy under the free vaccination policy in Guangzhou, in contrast to parents of girls in better health. This may be related to the perceived safety of the vaccine, which is a key factor influencing vaccine hesitancy (Tsui et al. 2023, Purvis et al. 2024). The government’s implementation of the free HPV vaccine program, along with health education and policy promotion in schools and communities, has contributed to enhancing parents’ confidence in the vaccine's safety.

A significant factor in HPV vaccine hesitancy among Chinese parents is the disparity in trust between domestically produced and imported vaccines. In this study, parents who expressed lower trust in domestic vaccines and preferred imported options had higher levels of hesitancy. Previous research on HPV vaccine awareness and willingness to vaccinate in China indicated that the public is willing to pay more for imported vaccines, especially older women (Zhang et al. 2021b).

This preference may be due to concerns about the safety and effectiveness of domestically produced vaccines. Factors such as vaccine-related information, concerns about side effects, and perceptions of vaccine safety and effectiveness also influence HPV vaccine uptake (Karafillakis et al. 2019, Purvis et al. 2024). In China, the acceptance of domestic HPV vaccines remains a challenge (Wong et al. 2020), and public distrust may be fueled by inadequate crisis management by vaccine companies (Yang et al. 2019). The results suggest that restoring confidence in domestic HPV vaccines remains a priority.

### Optimizing service delivery in community health centers

This study examines the factors influencing vaccine hesitancy within community health centers, guided by the theoretical framework of supply and demand adaptability (Liu et al. 2022). Specifically, it explores supply-side variables, focusing on the role of CHCs in vaccination services. The findings reveal that three key factors—parental satisfaction with healthcare centers, waiting time at these centers, and the ease of accessing information—are significant determinants of vaccine hesitancy scores.

First, the satisfaction with CHCs was found to be one of the influencing factors for vaccine hesitancy among parents of girls. Parental satisfaction with CHCs showed a positive correlation with vaccine hesitancy scores. Previous research has indicated that vaccine hesitancy is closely related to attitudes toward healthcare-related institutions, such as trust (Wemrell and Gunnarsson 2022, Krastev et al. 2023). In Guangzhou, where the free vaccination policy is implemented and local CHCs serve as vaccination sites, parents who expressed greater satisfaction with these centers were less likely to hesitate in vaccinating their daughters against HPV.

Second, parents who experienced waiting times over an hour reported lower vaccine hesitancy compared to those who waited of less than 10 minutes. This finding may highlight the positive influence of Guangzhou's free HPV bivalent vaccination program, suggesting that the policy encourages parents to be more willing to endure longer wait times for vaccination. However, this also should be interpreted considering potential social desirability bias. Parents may have underreported dissatisfaction with long wait times due to perceived expectations to endorse government-sponsored programs. This could partially explain the counterintuitive association between extended waiting periods and reduced vaccine hesitancy. While policy-driven tolerance remains a plausible explanation, future studies should employ mixed-methods approaches (e.g. in-depth interviews) to disentangle genuine acceptance from response bias.

Third, access to information through CHCs was another critical factor. Information scarcity is a key barrier to vaccine uptake (Holman et al. 2014, Karafillakis et al. 2019, Della Polla et al. 2020, Purvis et al. 2024). However, parents in this study who found it difficult to obtain relevant vaccination information were less hesitant. This may due to the concerted efforts of government, schools, and healthcare centers to provide free vaccination services and accessible information seemed to mitigate this issue, because parents’ vaccination intentions and information needs are closely linked to trust in institutions (Dubé et al. 2018). Parents who initially struggled to find information were more likely to trust the institutions involved, ultimately leading to reduced vaccine hesitancy. The collaborative role of these institutions in disseminating information proved effective in increasing parental confidence in vaccination.

### The role of family health in shaping vaccine hesitancy and acceptance

Family health also played a role in vaccine hesitancy, as indicated by a positive correlation between the family health scores and vaccine hesitancy scores. Parents with higher family health score—indicating stronger family health—were less hesitant to vaccinate their daughters against HPV. This suggests that parents who prioritize family health are more likely to view vaccination as an important preventive measure against HPV-related diseases. Consequently, health education efforts should emphasize the importance of vaccination in maintaining family health.

While previous studies have highlighted the impact of family health on children's well-being (Van Fossen et al. 2021) and healthcare utilization (García-Huidobro et al. 2012), particularly in primary healthcare settings, the data from this study suggest that the influence of family health on vaccine hesitancy may be more limited than expected. Further research is needed to better understand the complex relationship between family health and children's healthcare utilization, especially in the context of vaccine acceptance.

### Implications for low- and middle-income countries

Given the healthcare challenges faced by women in many low- and middle-income countries (LMICs), especially with a relatively culturally conservative background, it is challenging to expand HPV vaccination coverage for girls aged 9–15. This study offers key insights for LMICs.

First, the study indicated that agricultural communities should be targeted. Given the large agricultural populations in LMICs, vaccination and hesitancy in this group require continued attention and culturally sensitive in-depth research. A few randomized controlled trials have indicated that the effect of interventions focusing on vaccine education—such as videos (Zhu et al. 2022), mobile applications providing accessible HPV vaccine information (Shegog et al. 2022), and cervical cancer survivors' stories (Suzuki et al. 2022)—is controversial. Evidence-based tailored interventions specifically designed for farmers or low-income populations in LMICs remain scarce. This underscores the need for further context-specific research of factors impeding vaccination engagement in this population. Second, bridging confidence gaps in vaccines is important. Distrust in domestic bivalent vaccines (preference for imported 9-valent vaccines) is a barrier. LMICs that rely on cost-effective domestic vaccines should boost public confidence through science communication. Third, optimize service accessibility of vaccination spots. CHC service directly impacts uptake—satisfaction and information accessibility are key points. LMICs should enhance vaccination sites (e.g. simplifying information access) and strengthen school–community partnerships. In addition, the study provided potential evidence of policy-driven tolerance. LMICs may prioritize vaccine supply amid resource constraints while incrementally improving service efficiency.

### Limitations

This study's findings must be considered in light of its limitations. First, the reliance on convenience sampling may limit the generalizability of our findings, as the sample likely underrepresents parents with strong vaccine hesitancy or those less accessible through conventional recruitment channels. Consequently, these results may not fully reflect the broader spectrum of parental attitudes and contextual factors influencing vaccine decisions or attitudes. Future studies should aim to employ a random sampling method with more diverse participants to enhance the representativeness and robustness of the conclusions. Second, cross-sectional design prevents establishing causal relationships between the identified factors and vaccine hesitancy. Additionally, this research predominantly employed quantitative methods to assess vaccine hesitancy among parents of girls, which may not fully capture the nuanced factors influencing hesitancy. Integrating qualitative research, such as in-depth interviews, could provide richer insights into the underlying causes of vaccine hesitancy. Specifically, the study indicated the limited effect of family health. This allows for a more comprehensive understanding of the dynamics that shape parental vaccine acceptance. This would, in turn, inform the development of targeted interventions to reduce hesitancy.

## Conclusion

This study provides novel data on HPV vaccine hesitancy under a recently implemented free policy in a major Chinese city, providing evidence in literature from low-coverage regions. The identification of occupation-specific barriers and trust gaps provides actionable insights for policymakers worldwide, such as complementary strategies to immunization on the spot or parenting interventions, particularly in contexts where public health systems seek to balance free access with cultural and socio-economic challenges.

The results indicate that, compared to other categories of vaccine hesitancy in China, the proportion of parents highly hesitant about the HPV vaccine in Guangzhou was relatively low, likely due to the impact of the free vaccination policy. However, significant room for improvement remains. Key factors influencing vaccine hesitancy include parental occupation, differences in trust between domestic and imported vaccines, and family health functioning, particularly the health of the girls. Furthermore, hesitancy was largely influenced by the quality of services provided by CHCs, such as waiting times, ease of access to information, and overall satisfaction. Interestingly, the study found that hesitancy was lower among parents of girls in average health and those who experienced longer waiting times at healthcare centers, potentially due to the influence of the free HPV vaccination policy.

## Supplementary Material

czaf087_Supplementary_Data

## Data Availability

The datasets generated and/or analyzed during the current study are not publicly available due privacy protection but are available from the corresponding author on reasonable request.

## Reflexivity statement

The authors are all females and span multiple levels of seniority in China. One of the authors is a long-time expert in the field of behavioral public health and health policy evaluation. One author has experience with women's and children's research on cervical and breast cancer. Three authors have field experience and have been adequately trained for the specific field research of this study.
