# Supplementary material for: Understanding determinants of parental HPV vaccine hesitancy under a municipal free vaccination program in Guangzhou, China
Source: Health Policy Plan. 2025 Nov 10;41(2):176–85. doi: 10.1093/heapol/czaf087 (PMC12906768; doi:10.1093/heapol/czaf087)
Supplement: czaf087_Supplementary_Data [file czaf087_supplementary_data.docx]

Appendix Table 1. Measurement of relevance, appropriateness, accessibility, and quality based on supply-demand alignment theory

|  |  | Item |
| --- | --- | --- |
| Relevance | If the supply of HPV vaccines / related information provision meets the actual needs of parents |  |
|  |  | Do you think this service (free bivalent HPV vaccination for girls) meets your actual needs? |
|  |  | Does the information promotion of “free bivalent HPV vaccination” meet your information needs? |
|  |  | If you need to access information related to “free bivalent HPV vaccination,” which sources do you prefer? |
| Appropriateness | If the HPV vaccination service considers the diversity and complexity of children's vaccination |  |
|  |  | Is a personalized platform provided for inquery or appointments for HPV vaccinations and related information to flexibly response complex appointments/vaccination situations? |
| Accessibility | Accessibility of HPV vaccination or related information |  |
|  |  | How far away from you is the free HPV vaccination service acceptable? |
|  |  | Is there a vaccination site near your place of residence? |
|  |  | Do you think accessing “free bivalent HPV vaccination” information about through community health centers/schools is convenient and easy? |
| Quality | Services quality related to HPV vaccination |  |
|  |  | How long is the average waiting time in the queue for you to have your child vaccinated? |
|  |  | Do you think the current management system and process of the vaccination service at the CHC is sound? |
|  |  | How satisfied are you with the vaccination service at CHC? (0~100 sliding scale) |

Appendix Table 2. Comparison of parents’ VHS scores with different characteristics (n=411)

| **Variables** | **Groups** | **n(%)** | **M(P25,P75)** | **β (95% CI)** | **P value** |
| --- | --- | --- | --- | --- | --- |
| **If recommended by doctors** |  |  |  |  |  |
|  | No | 180(43.79) | 36.00(33.00,38.00) | Reference | N/A |
|  | Yes | 231(56.20) | 37.00(34.00,40.00) | 0.871(-0.155,1.897) | 0.096 |
| **Prior vaccine refusal** |  |  |  |  |  |
|  | No | 341(82.96) | 36.00(34.00,39.00) | Reference | N/A |
|  | Yes | 70(17.03) | 35.00(30.00,39.25) | -1.586(-2.936,-0.236) | 0.021* |
| **Previous unsatisfactory vaccine experience** |  |  |  |  |  |
|  | No | 306(74.45) | 36.00(34.00,39.25) | Reference | N/A |
|  | Yes | 105(25.54) | 36.00(32.00,38.00) | -1.390(-2.554,-0.226) | 0.019* |
| **Worried about side effects** |  |  |  |  |  |
|  | Very concerned | 93(22.38) | 35.00(33.00,38.00) | -0.503(-0.613,0.382) | 0.536 |
|  | Somewhat concerned | 242(58.88) | 36.00(34.00,39.00) | 0.742(-0.613,2.097) | 0.283 |
|  | Not concerned | 76(18.49) | 38.00(31.25,41.75) | Reference | N/A |
| **Health status of the girls** |  |  |  |  |  |
|  | Poor | 12(2.91) | 36.00(33.00,39.00) | Reference | N/A |
|  | Moderate | 87(21.16) | 36.00(34.00,39.00) | 4.175(1.016,7.335) | 0.010* |
|  | Good | 312(75.91) | 33.00(23.50,39.25) | 4.311(1.293,7.329) | 0.005* |
| **Trust in domestic versus imported vaccines** |  |  |  |  |  |
|  | High level of trust (no preference for imported vaccines) | 97(23.60) | 38.00(34.00,41.00) | Reference | N/A |
|  | Mostly trust (some concerns about certain vaccines) | 228(55.47) | 36.00(34.00,38.00) | -1.087(-2.33,0.155) | 0.086 |
|  | Not very trusting (preference for imported vaccines) | 77(18.73) | 36.00(32.00,38.50) | -2.118(-3.683,-0.554) | 0.008* |
|  | Very distrustful (no preference for domestically produced vaccines) | 9(2.18) | 35.00(29.00,39.00) | -3.255(-6.827,0.316) | 0.074 |
| **Attention to HPV vaccine-related information** |  |  |  |  |  |
|  | Lack of attention | 41(9.97) | 36.00(33.00,38.00) | Reference | N/A |
|  | Limited attention | 109(26.52) | 35.00(33.00,37.00) | 0.913(-0.959,2.785) | 0.339 |
|  | Moderate attention | 170(41.36) | 35.00(33.00,39.00) | 1.581(-0.197,3.359) | 0.081 |
|  | Significant attention | 91(22.14) | 38.00(35.00,42.00) | 2.892(0.970,4.814) | 0.003* |
| **Adequacy of HPV vaccine service in meeting actual needs** |  |  |  |  |  |
|  | No | 107(26.03) | 35.00(33.00,38.00) | Reference | N/A |
|  | Yes | 304(73.96) | 36.00(34.00,40.00) | 1.135(-0.024,2.294) | 0.055 |
| **Adequacy of HPV vaccine publicity in meeting information needs** |  |  |  |  |  |
|  | No | 124(30.17) | 35.00(32.00,38.00) | Reference | N/A |
|  | Yes | 287(69.82) | 37.00(34.00,40.00) | 1.179(0.071,2.286) | 0.037* |
| **Distance of CHC^a^** |  |  |  |  |  |
|  | ≥5km | 54(13.13) | 36.00(33.00,37.25) | Reference | N/A |
|  | 3-4.9km | 97(23.60) | 36.00(33.00,39.00) | 0.533(-1.221,2.287) | 0.551 |
|  | 1-2.9km | 167(40.63) | 36.00(33.00,40.00) | 0.386(-1.231,2.003) | 0.640 |
|  | ＜1km | 93(22.62) | 36.00(34.00,40.00) | 1.202(-0.565,2.969) | 0.183 |
| **Proximity to vaccination locations** |  |  |  |  |  |
|  | No | 116(28.22) | 35.00(32.00,38.00) | Reference | N/A |
|  | Yes | 295(71.77) | 37.00(34.00,40.00) | 1.999(0.881,3.118) | <0.001* |
| **Waiting Time at CHC^a^** |  |  |  |  |  |
|  | ＜10 minutes | 35(8.51) | 36.00(31.00,39.00) | Reference | N/A |
|  | 10-29 minutes | 174(42.33) | 36.50(34.00,40.00) | 2.999(1.105,4.893) | 0.002* |
|  | 30-59 minutes | 153(37.22) | 36.00(33.00,38.50) | 1.988(0.073,3.904) | 0.042* |
|  | ＞1 hour | 49(11.92) | 36.00(33.50,39.00) | 1.967(-0.296,4.230) | 0.088 |
| **Difficulty in accessing information from the school** |  |  |  |  |  |
|  | Very easy | 74(18.00) | 38.00(34.00,43.00) | Reference | N/A |
|  | Relatively easy | 208(50.60) | 36.00(34.00,38.00) | -2.050(-3.434,-0.667) | 0.004* |
|  | Relatively difficult | 103(25.06) | 36.00(33.00,39.00) | -2.086(-3.644,-0.529) | 0.009* |
|  | Very difficult | 26(6.32) | 36.00(31.00,38.25) | -3.017(-5.347,-0.686) | 0.011* |
| **Difficulty in accessing information from CHC ^a^** |  |  |  |  |  |
|  | Very easy | 92(22.38) | 38.00(34.00,42.00) | Reference | N/A |
|  | Relatively easy | 215(52.31) | 36.00 (34.00,38.00) | -1.742(-3.021,-0.463) | 0.008* |
|  | Relatively difficult | 83(20.19) | 36.00(32.00,39.00) | -1.433(-2.988,0.121,) | 0.071 |
|  | Very difficult | 21(5.10) | 38.00(32.00,40.50) | -1.242(-3.725,1,241) | 0.327 |
| **Understanding of the vaccination process** |  |  |  |  |  |
|  | No | 208(50.60) | 35.00(33.00,38.00) | Reference | N/A |
|  | Yes | 203(49.39) | 37.00(34.00,41.00) | 1.063(0.046,2.079) | 0.040* |
| **The soundness of the management system in CHC^a^** |  |  |  |  |  |
|  | No | 192(46.71) | 35.00(33.00,38.00) | Reference | N/A |
|  | Yes | 219(53.28) | 37.00(34.00,41.00) | 1.730(0.720,2.740) | <0.001* |
| **Provision of personalized and comprehensive platforms for reservations and inquiries** |  |  |  |  |  |
|  | No | 210(51.09) | 36.00(33.00,38.00) | Reference | N/A |
|  | Yes | 201(48.90) | 37.00(34.00,40.00) | 1.026(0.008,2.043) | 0.048* |
| **Preferred sources of HPV vaccine-related information** |  |  |  |  |  |
|  | Healthcare professionals | 95(23.11) | 37.00(34.00,41.00) | Reference | N/A |
|  | Girl's school | 78(18.97) | 35.00(32.75,38.00) | -1.082(-2.656,0.492) | 0.178 |
|  | CHC^a^ | 86(20.92) | 36.50(34.00,41.00) | 0.146(-1.387,1.679) | 0.853 |
|  | Official public accounts or websites | 80(19.46) | 36.00(33.00,39.00) | -0.230(-1.793,1.333) | 0.773 |
|  | Others | 72(18.00) | 36.00(34.00,38.00) | -1.214(-2.823,0.396) | 0.139 |
| **CHC ^a^ satisfaction** | - | - | - | 0.076(0.052,0.100) | <0.001* |
| **Family health score** | - | - | - | 0.343(0.262,0.424) | <0.001* |

**^a^** CHC: community healthcare centers

*P＜0.05

Appendix Table 3. Generalized linear regression of demographic and socialeconomic factors associated with the HPV vaccine hesitancy among parents of girls (n=411)

| **Variables** | | **β(SE)** | **95%CI** | **X^2^(df)** | **P value** |
| --- | --- | --- | --- | --- | --- |
| **Gender** (reference: Male) |  |  |  |  |  |
|  | Female | 1.03(0.56) | (-0.07,2.13) | 3.35(1) | 0.07 |
| **Education** (reference: Primary school and below) |  |  |  |  |  |
|  | Middle school/high school/vocational school | 2.40(1.25) | (-0.06,4.85) | 3.65(1) | 0.32 |
|  | Undergraduate or college | 2.59(1.27) | (0.11,5.08) | 4.17(1) | 0.27 |
|  | Postgraduate or above | 3.59(1.54) | (0.57,6.6) | 5.43(1) | 0.02* |
| **Occupation category** (reference: Enterprise/Business/Service industry) |  |  |  |  |  |
|  | Government civil servants | 0.85(0.98) | (-1.07,2.77) | 0.75(1) | 0.93 |
|  | Worker | 1.03(1) | (-0.92,2.99) | 1.07(1) | 0.03* |
|  | Farmer | -4.34(1.82) | (-7.91,-0.76) | 5.65(1) | 0.23 |
|  | Public institution employee | -0.33(0.72) | (-1.74,1.07) | 0.22(1) | 0.30 |
|  | Others | -0.26(0.71) | (-1.65,1.13) | 0.14(1) | 0.71 |

*: P＜0.05

Appendix Table 4. Generalized linear regression of demographic and socialeconomic factors and health state indicators associated with the HPV vaccine hesitancy among parents of girls (n=411)

| **Variables** | | **β(SE)** | **95%CI** | **X^2^(df)** | **P value** |
| --- | --- | --- | --- | --- | --- |
| **Gender** (reference: Male) |  |  |  |  |  |
|  | Female | 0.37(0.53) | (-0.68,1.41) | 0.47(1) | 0.49 |
| **Education** (reference: Primary school and below) |  |  |  |  |  |
|  | Middle school/high school/vocational school | 2.28(1.18) | (-0.03,4.58) | 3.74(1) | 0.05* |
|  | Undergraduate or college | 2.01(1.19) | (-0.33,4.35) | 2.83(1) | 0.09 |
|  | Postgraduate or above | 2.44(1.46) | (-0.42,5.29) | 2.8(1) | 0.09 |
| **Occupation category** (reference: Enterprise/Business/Service industry) |  |  |  |  |  |
|  | Government civil servants | 0.9(0.93) | (-0.93,2.73) | 0.93(1) | 0.33 |
|  | Worker | 0.99(0.93) | (-0.84,2.82) | 1.13(1) | 0.29 |
|  | Farmer | -2.84(1.72) | (-6.21,0.53) | 2.73(1) | 0.10 |
|  | Public institution employee | 0.06(0.67) | (-1.26,1.38) | 0.01(1) | 0.93 |
|  | Others | -0.01(0.67) | (-1.32,1.3) | 0(1) | 0.99 |
| **Health State of girls** (reference: Good) |  |  |  |  |  |
|  | Moderate | 0.73(0.6) | (-0.44,1.9) | 1.5(1) | 0.22 |
|  | Poor | -1.59(1.5) | (-4.53,1.35) | 1.12(1) | 0.29 |
| **Family health score** |  | 0.32(0.04) | (0.23,0.4) | 54.2(1) | ＜0.01* |

*: P＜0.05

Appendix Table 5. Generalized linear regression of demographic and socialeconomic factors, health state indicators, and attitude associated with the HPV vaccine hesitancy among parents of girls (n=411)

| **Variables** | | **β(SE)** | **95%CI** | **X^2^(df)** | **P value** |
| --- | --- | --- | --- | --- | --- |
| **Gender** (reference: Male) |  |  |  |  |  |
|  | Female | 0.33(0.54) | (-0.72,1.38) | 0.38(1) | 0.54 |
| **Education** (reference: Primary school and below) |  |  |  |  |  |
|  | Middle school/high school/vocational school | 2.2(1.17) | (-0.09,4.49) | 3.53(1) | 0.06 |
|  | Undergraduate or college | 1.99(1.19) | (-0.34,4.33) | 2.79(1) | 0.10 |
|  | Postgraduate or above | 2.55(1.46) | (-0.31,5.42) | 3.06(1) | 0.08 |
| **Occupation category** (reference: Enterprise/Business/Service industry) |  |  |  |  |  |
|  | Government civil servants | 0.84(0.92) | (-0.97,2.65) | 0.83(1) | 0.36 |
|  | Worker | 1.09(0.92) | (-0.72,2.9) | 1.39(1) | 0.24 |
|  | Farmer | -2.85(1.71) | (-6.2,0.5) | 2.77(1) | 0.10 |
|  | Public institution employee | 0.12(0.68) | (-1.21,1.45) | 0.03(1) | 0.86 |
|  | Others | -0.18(0.66) | (-1.48,1.11) | 0.08(1) | 0.78 |
| **Prior vaccine refusal** (reference: No) |  |  |  |  |  |
|  | Yes |  |  |  |  |
| **Previous unsatisfactory vaccine experience** (reference: No) |  |  |  |  |  |
|  | Yes |  |  |  |  |
| **Health State of girls** (reference: Good) |  |  |  |  |  |
|  | Moderate | 0.92(0.59) | (-0.24,2.08) | 2.41(1) | 0.12 |
|  | Poor | -1.18(1.5) | (-4.12,1.76) | 0.62(1) | 0.43 |
| **Prior vaccine refusal** (reference: No) |  |  |  |  |  |
|  | Yes | 0.8(0.67) | (-0.5,2.11) | 1.46(1) | 0.23 |
| **Previous unsatisfactory vaccine experience** (reference: No) |  |  |  |  |  |
|  | Yes | 0.57(0.58) | (-0.57,1.72) | 0.97(1) | 0.32 |
| **Trust in domestic versus imported vaccines** (reference: High level of trust (no preference for imported vaccines)) |  |  |  |  |  |
|  | Mostly trust (some concerns about certain vaccines) | -1.04(0.59) | (-2.19,0.12) | 3.1(1) | 0.08 |
|  | Not very trusting (preference for imported vaccines) | -1.63(0.76) | (-3.11,-0.14) | 4.61(1) | 0.03* |
|  | Very distrustful (no preference for domestically produced vaccines) | -0.72(1.71) | (-4.07,2.64) | 0.18(1) | 0.68 |
| **Attention to HPV vaccine-related information**  (reference: Lack of attention) |  |  |  |  |  |
|  | Limited attention | -1.04(0.59) | (-2.19,0.12) | 3.1(1) | 0.08 |
|  | Moderate attention | -1.63(0.76) | (-3.11,-0.14) | 4.61(1) | 0.03* |
|  | Significant attention | -0.72(1.71) | (-4.07,2.64) | 0.18(1) | 0.68 |
| **Family health score** |  | 0.3(0.04) | (0.21,0.39) | 46.96(1) | ＜0.01* |

*: P＜0.05
